# Shared and Unique Structural Covariance Connectivity in Comorbidity of Obsessive-Compulsive Disorder and Major Depressive Disorder

**DOI:** 10.1155/da/1087782

**Published:** 2025-11-26

**Authors:** Hongyu Du, Feng Gao, Yan Han, Chuman Xiao, Qian Li, Douyu Zhang, Zhiyan Wang, Qianmei Yu, Sainan Cai, Jie Fan, Xiongzhao Zhu

**Affiliations:** ^1^Medical Psychological Center, The Second Xiangya Hospital, Central South University, Changsha, Hunan 410011, China; ^2^Medical Psychological Institute of Central South University, Changsha, Hunan 410011, China; ^3^National Clinical Research Center for Mental Disorders, Changsha, Hunan 410011, China; ^4^Department of Radiology, The Second Xiangya Hospital, Central South University, Changsha, Hunan 410011, China

**Keywords:** comorbidity, major depressive disorder, obsessive–compulsive disorder, structural covariance network

## Abstract

**Background:**

The comorbidity of obsessive–compulsive disorder (OCD) and major depressive disorder (MDD) is a prevalent clinical phenomenon, which is associated with greater symptom severity, suicide risk, and poorer treatment outcomes. However, the neural basis of this comorbidity remains unclear. The aim of this study was to investigate the common and unique neural basis of comorbidity compared with OCD and MDD alone.

**Methods:**

A total of 67 patients with comorbid OCD and MDD, 89 patients with OCD alone, 94 patients with MDD alone, and 94 healthy controls (HCs) completed the acquisition of T1-weighted structural images and were included in the present study. The gray matter volume of each brain region in the AAL116 atlas was calculated, based on which the structural covariance between each pair of brain regions was measured. One-way ANCOVA were performed to explore the structural covariance differences among the four groups.

**Results:**

Compared with HC, patients with comorbidity and patients with OCD or MDD alone showed some common altered structural connections (*p* < 0.05, false discovery rate [FDR] correction). Patients with comorbidity showed unique altered correlation strength of gray matter volume between left cerebelum_crus1 and left orbital frontal cortex (OFC), left Rolandic operculum, right rectus, right parahippocampal gyrus, left and right fusiform gyrus (FG), left Heschl gyrus, left and right superior temporal gyrus (STG), and left cerebelum_crus2; these 10 connections were also significantly different when comparing comorbidity with OCD and MDD separately (*p* < 0.05, FDR correction).

**Conclusions:**

Compared with OCD or MDD alone, comorbidity showed both common and unique altered structural covariant connections of gray matter structure. The unique structural connections observed in comorbidity were concentrated between the cerebellum and other brain regions. These findings highlight the crucial role of the cerebellum in the neural basis of comorbid OCD and MDD.

## 1. Introduction

The comorbidity of obsessive–compulsive disorder (OCD) and major depressive disorder (MDD) is a common clinical phenomenon. The lifetime prevalence of MDD in patients with OCD is ~62.7%–78.2% [1, 2], and obsessions or compulsions occur in about 22%–38% of patients with MDD [3, 4]. Compared with OCD or MDD alone, comorbidity exhibits important clinical features, including an elevated risk of suicide attempts [5, 6], functional disability, increased symptom severity, and poorer treatment outcome [7, 8]. Despite its clinical significance, the neural mechanisms underlying the comorbidity remain unclear.

Previous studies usually investigated the neural mechanisms underlying OCD or MDD alone. For example, regarding the neural mechanisms of OCD, studies have consistently found structural and functional abnormalities in the cortico–striatal–thalamo–cortical (CSTC) circuitry [9]. The CSTC circuit can be further subdivided into three sub-circuits: the ventral emotional circuit, the ventral cognitive circuit, and the dorsal cognitive circuit [10]. In addition to the CSTC circuit, some other circuit abnormalities are also integrated into the neural mechanism of OCD, such as the sensorimotor circuit and the prefrontal-limbic circuit [11–13]. In terms of MDD, the pathological mechanism is mainly related to abnormalities of the fronto-limbic network and the reward circuitry [14, 15].

Several studies have also tried to explore the neural underpinnings of the comorbidity of OCD and MDD. However, the results from these studies have been inconsistent. For example, Cardoner et al. [16] found decreased gray matter volume in the medial orbitofrontal cortex and medial frontal gyrus in patients with comorbid OCD and MDD, as well as in patients with OCD alone. This finding suggested that the comorbidity may share a common neurological basis with each individual disorder. However, another study found that comorbidity may have distinct neurological basis compared to single diagnostic disease. Specifically, Saxena et al. [17] used positron emission tomography (PET) and found that the brain metabolic abnormalities, such as hypermetabolism in the thalamus and right caudate, were shown in patients with OCD or MDD alone, while not in patients with comorbid OCD and MDD, supporting the distinct neural alterations in the comorbidity. This inconsistency may be also parallel to previous views on the comorbidity mechanism. That is, some researchers considered that MDD is secondary to the functional impairment caused by OCD [18, 19], and therefore, comorbidity may be the superposition or commonality of the pathological mechanisms of OCD and MDD. While other researchers argued that there is no causal relationship between OCD and MDD, and the occurrence of comorbidity may be independent of OCD or MDD alone [20, 21], which call for the distinct neural underpinnings in commodity OCD and MDD. The discrepancies observed in the current neuroimaging findings may obscure the answer of the mechanism of comorbidity. These discrepancies may be attributed to several potential factors in the previous studies. One significant factor may be that the samples in previous studies only met the diagnostic criteria for OCD with concurrent depressive symptoms, rather than patients with current comorbid OCD and MDD [22]. As a result, these findings may not accurately reflect the neural basis of comorbidity. Additionally, the relatively small sample size in some studies may also be a limitation. Hence, the neural basis of comorbidity still remains unclear, and it is yet to be determined whether it is common or unique to OCD or MDD alone.

Recently, the use of structural covariance network (SCN) to evaluate brain connectivity has emerged as a powerful tool to study the human brain. SCN is a method for describing regional volume covariation between brain regions with common development/maturation trajectories [23, 24]. Structural covariance between regions is hypothesized to originate from developmental processes [25], to be influenced by brain-derived neurotrophic factor and activity-dependent structural plasticity [26, 27], and to measure brain connectivity properties on a larger time scale that represent trait-like (stable or lasting) features [25]. Mental disease is often a rather complex and protracted process, and the comorbidity may be even more complex and prolonged. The SCN can reflect more persistent and stable brain changes and may be more conducive to the interpretation of the neural mechanisms of comorbidity. SCN has been widely used in psychiatric research, including the studies of OCD and MDD. For example, one study found that OCD patients exhibited greater structural covariance between the left ventral-rostral putamen and the left inferior frontal gyrus/frontal operculum region compared to healthy controls (HCs) [28]. Another study by Zhang et al. [29] showed that the thickness covariance of the granular (refers to dense neurons like particles) and dysgranular insula was different in OCD patients compared to controls. One study using SCN to investigate the network features of MDD revealed significant differences in the salience network (brain network detecting salient stimuli, guiding attention/responses), medial temporal lobe network, default mode network, and central executive network between MDD and HCs [30]. Han et al. [31] also found that patients with MDD had altered structural covariance of the nucleus accumbens (NAcc), which is connected to key brain regions in the reward system, including the medial orbitofrontal cortex, amygdala, insula, parahippocampal gyrus, precuneus, thalamus, hippocampus, and cerebellum. However, to our knowledge, no studies have yet examined the characteristics of SCN in the comorbidity of OCD and MDD.

Taken together, the aim of this study was to investigate the possible neural underpinnings of the comorbidity of OCD and MDD, and to determine whether the neural underpinnings of the comorbidity show common or unique alterations to OCD or MDD alone. Patients with comorbid OCD and MDD were included as subjects, and we included patients with OCD alone, patients with MDD alone, and healthy individuals as controls. This study analyzed the characteristic of SCN for each participant, and given the viewpoints in previous literature that comorbidity may be the commonality or superposition of OCD and MDD alone or it may be independent of OCD or MDD alone, we hypothesized that SCN alterations in comorbidity of OCD and MDD show not only commonalities but also uniqueness compared to OCD or MDD alone.

## 2. Methods

### 2.1. Participants

A total of 263 patients with MDD alone, OCD alone, and OCD and MDD comorbidity were recruited from the psychological clinic at Second Xiangya Hospital of Central South University. Two experienced psychiatrists conducted diagnoses and comorbidity assessments using the Structured Clinical Interview for the DSM-5 (SCID). Exclusion criteria were: (1) other psychiatric disorders other than OCD or MDD; (2) a history of major medical or neurological problems (such as cerebral infarction, cerebral hemorrhage, coronary heart disease, and severe traumatic brain injury, etc.); and (3) any contraindications to MRI scanning. Seven patients with OCD and six patients with MDD were excluded from statistical analyses due to the bad image quality. Of the included 250 patients, 101 were under treatment with medication (SSRI: *n* = 84; SNRI: *n* = 7; SSRI + atypical antipsychotic: *n* = 10), and 149 were drug-naive or drug-free for at least 3 months prior to the MRI scan.

One hundred and three healthy individuals were recruited from local colleges and communities in Changsha through advertisement. Exclusion criteria were: (1) any psychiatric disorders; (2) a history of major medical or neurological problems; and (3) any contraindications to MRI scanning. Of these subjects, nine were excluded due to bad image quality, and the remaining 94 healthy individuals formed the HC group.

Patients and HCs were all right-handed, had at least 9 years of formal education, and were aged between 18 and 35. Using the G-POWER software, with an effect size of 0.25 and a power of 0.9, the total minimum sample size is required to be 232. Our actual sample size was 344, which guaranteed the statistical power. All participants provided informed consent, and the study was approved by Ethics Committee of the Second Xiangya Hospital of Central South University.

### 2.2. Clinical Assessment

After diagnosis, each participant was given a semi-structured interview conducted by a psychiatrist, to collect demographic information and clinical variables. The Beck Depression Inventory (BDI-I) [32] and the State Trait Anxiety Inventory (STAI) [33] were used to assess participants' levels of depression and anxiety. The STAI consists of the State Anxiety Subscale and the Trait Anxiety Subscale, each of which has 20 items, rated on a scale of 1–4. OCD symptom severity was evaluated using the Yale-Brown Obsessive-Compulsive Scale (Y-BOCS) [34]. The first five items of the Y-BOCS assessed the severity of obsessions and the last five items assessed the severity of compulsions. Each item was scored on a 5-point scale ranging from 0 to 4. Verbal intelligence was assessed by summing the standard scores of the four verbal subtests (knowledge, arithmetic, similarity, and digit span) of the Wechsler Adult Intelligence Scale-Revised for China (WAIS-RC).

### 2.3. Scan Acquisition

Imaging data were acquired on a Siemens Skyra 3.0-T MRI scanner at the Second Xiangya Hospital of Central South University. T1-weighted structural images of the whole brain were obtained using three-dimensional magnetization-prepared rapid gradient echo (MPRAGE), with the following main parameters: 1900-ms repetition time, 2.01-ms echo time, 176 slices, 1.00-mm slice thickness, 1.0 × 1.0 × 1.0-mm voxel size, 9° flip angle, 900-ms inversion time, 256-mm field of view, and 256 × 256 matrix.

Before the scan, we will provide detailed instructions. And we used noise-cancelling headphones and wedge-shaped soft pads to fix the patient's head, and inserted some sponge between the head and the slot to further prevent the head from shaking, in order to ensure the quality of the scan images.

### 2.4. Image Preprocessing and VBM Analyses

Image preprocessing was performed using the CAT12 toolbox (http://dbm.neuro.uni-jena.de/cat12/). The standard pipeline steps were adopted including coregistration, spatial normalization using the DARTEL (Diffeomorphic Anatomical Registration Through Exponentiated Lie Algebra) strategy, segmentation, resampling (1.5 mm × 1.5 mm × 1.5 mm), nonlinear modulation, and image quality inspection. Finally, the gray matter maps were smoothed using 6 mm full width at half maximum Gaussian kernel (FWHM). Total intracranial volume (TIV) for each participant was calculated.

### 2.5. SCN Construction

We defined 116 seed regions of the whole brain by using the AAL116 atlas, which contains 90 seeds in the cerebrum and 26 in the cerebellum. The gray matter volume within each seed region was calculated, based on which, the covariance between the average gray matter volumes of any two seed regions was computed. Specifically, each connection in each group was computed using following model [31]:

  $$\begin{array}{c} V_{i}=\beta 1\times G_{\text{HC}}+\beta 2\times G_{\text{MDD}}+\beta 3\times G_{\text{OCD}}+\beta 4\times G_{\text{comorbidity}}+\beta 5\times V_{j}\times G_{\text{HC}}+\beta 6\times V_{j}\times G_{\text{MDD}}+\beta 7\times V_{j}\times G_{\text{OCD}}+\beta 8\times V_{j}\times G_{\text{comorbidity}}+\beta 9\times \text{control\_variables (TIV/sex/age/intelligence)}. \end{array}$$

In this model, *V*_*j*_ (*j =* 1,2,…116) was the mean gray matter volumes of each seed and *V*_*i*_ (*i*, *j =* 1,2,…116) was the mean gray matter volume of 115 remaining brain regions. *G*_HC_, *G*_MDD_, *G*_OCD_, and *G*_comorbidity_ were variables represent different groups. Take *G*_HC_ for example, when a subject belongs to HC group, the *G*_HC_ would be coded as 1, otherwise as 0. Then, *β5*, *β6*, *β7*, and *β8* were, respectively, the connections between *V*_*i*_ and *V*_*j*_ of the SCN in HC group, MDD group, OCD group, and comorbidity group. TIV, sex, age, and intelligence were controlled as variables of no interest. The SCN in this study contained a total of 6670 (*C*_116_^2^) connections.

### 2.6. Statistical Analysis

The above model was built in SPM12 with one-way ACNOVA design. F contrast between *β5*, *β6*, *β7*, and *β8 was set* to compare each connection of SCN among the four groups (*p* < 0.001, uncorrected), with age, sex, and verbal intelligence designated as covariates. For the connection which was significant in the *F* contrast, post hoc *t* contrasts were then set to identify differences between HC and each of the three patient groups (*β5* vs. *β6*, *β5* vs. *β7*, *β5* vs. *β8*). The connections of SCN were treated as unique altered connections of SCN in patients with comorbidity if *t* contrasts between HC group and comorbidity group (*β5* vs. *β8*) were significant while *t* contrast between HC group and MDD group (*β5* vs. *β6*), and HC group and OCD group (*β5* vs. *β7*) *were not significant*. These unique altered connections of SCN in patients with comorbidity would be compared in comorbidity vs. OCD (*β8* vs. *β7*) and comorbidity vs. MDD (*β8* vs. *β6*), respectively, to further confirm whether these connections are unique neuroimaging alterations of the comorbidity. All post hoc *t*-tests were controlled for age, sex, and verbal intelligence, and *t*-tests between patient groups were further controlled for medication status by adding the variable of medication status to the model. All post hoc *t*-tests were conducted with a threshold of *p* < 0.05 and corrected for false discovery rate (FDR) correction. FDR correction was conducted separately within each pairwise comparison.

We conducted the Kolmogorov–Smirnov test (KS) test on the normality and Levene test on the homogeneity of variance of the demographic and clinical variables, and the results are shown in Supporting Information Tables S1 and S2. For continuous variables which didn't satisfy the normal distribution or homogeneity of variance, we conducted the Kruskal–Wallis test (for more than two groups) and the Mann–Whitney test (for two groups); Otherwise, the *F* test or the *t* test was adopted. For the categorical variables, Chi-squared tests were used.

## 3. Results

### 3.1. Demographic and Clinical Variables

As shown in Table 1, our study included 67 patients with comorbid OCD and MDD, 89 patients with 109 OCD alone, 94 patients with MDD alone, and 94 healthy individuals. There were no significant differences in age and verbal intelligence between the four groups. The percentage of female subjects in MDD was higher than in OCD (*χ*^2^ (1) = 11.30, *p* = 0.01, *φ =* 0.25).

No significant differences were found in terms of age-onset and medication between the three patient groups, and there were no significant differences in Y-BOCS, YBOCS-C, and YBOCS-O scores between OCD and comorbidity. *F* test of TIV showed significant differences (*F* (3, 340) = 5.30, *p* < 0.001, *η*^2^ *=* 0.45). TIV was higher in OCD and HC than in MDD (*t* (181) = 3.86, *p* < 0.001, *d =* 0.57; *t*(186) = 2.84, *p* = 0.005, *d =* 0.41). Kruskal–Wallis test of the scores of BDI, STAI-T, and STAI-S showed significant differences (H (3) = 212.34, *p* < 0.001, *η*^2^ = 0.63; H(3) = 177.41, *p* < 0.001, *η*^2^ *=* 0.52; H(3) = 150.02, *p* < 0.001, *η*^2^ *=* 0.44). BDI score were higher in three patient groups (OCD, MDD, Comorbidity) than in HC (*U* = 1091.00, *p* < 0.001, *r = −*0.64; *U* = 232.50, *p* < 0.001, *r =* −0.82; *U* = 132.50, *p* < 0.001, *r =* −0.81), STAI-T score were higher in three patient groups (OCD, MDD, Comorbidity) than in HC (*U* = 779.00, *p* < 0.001, *r =* −0.70; *U* = 321.50, *p* < 0.001, *r =* −0.80; *U* = 243.50, *p* < 0.001, *r =* −0.79), STAI-S score were higher in three patient groups (OCD, MDD, Comorbidity) than in HC (*U* = 1299.00, *p* < 0.001, *r =* −0.59; *U* = 539.50, *p* < 0.001, *r =* −0.76; *U* = 415.00, *p* < 0.001, *r =* −0.74), BDI score were higher in MDD and comorbidity than in OCD (*U* = 1345.00, *p* < 0.001, *r =* −0.59; *U* = 812.50, *p* < 0.001, *r =* −0.62), STAI-T score were higher in MDD and comorbidity than in OCD (*U* = 2799.00, *p* < 0.001, *r =* −0.29; *U* = 1654.50, *p* < 0.001, *r =* −0.38), STAI-S score were higher in MDD and comorbidity than in OCD (*U* = 2760.50, *p* < 0.001, *r =* −0.29; *U* = 1866.00, *p* < 0.001, *r =* −0.32).

### 3.2. Intergroup Comparison of SCN

One-way ANCOVA of SCN revealed 36 structural connections with statistically significant differences between the four groups (Supporting Information Table S3).

Within the mask of initial ANCOVA result, post-hoc *t*-tests revealed 15 connections with statistically significant differences between comorbidity and HC, 11 connections between OCD and HC, 11 connections between MDD and HC (Table 2). All results above were *p* < 0.05 with FDR correction. And the uncorrected results are shown in Supporting Information Table S4.

**Figure 1:**
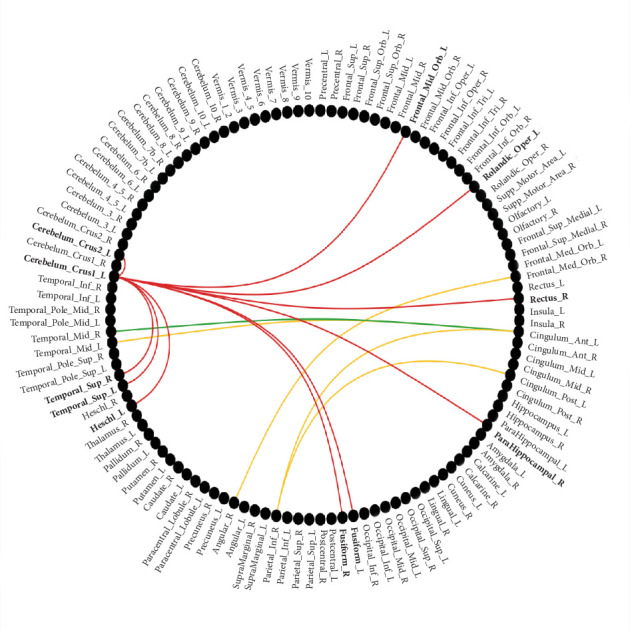
Post-hoc *t*-test of SCN between comorbidity and HC. Notes: There were 15 connections with significant differences, all *p* < 0.05 with FDR correction. Red line: altered connections unique to the comorbidity; green line: altered connections shared by three groups; yellow line: altered connections shared by comorbidity and MDD; altered connections shared by comorbidity and OCD were not found.

Altered connections shared by three groups were increased right middle temporal gyrus (MTG)-left anterior cingulate gyrus connection (*t*(159) *=* 3.61, *p* = 0.003; *t*(181) = 2.73, *p* = 0.008; *t*(186) = 3.11, *p* = 0.003), see green line in Figure 1; Altered connections shared by comorbidity and MDD were increased right inferior parietal lobe-left anterior cingulate gyrus connection (*t*(159) = 2.96, *p* = 0.007; *t*(186) = 3.74, *p* = 0.001), increased right inferior parietal lobe-left posterior cingulated gyrus connection (*t*(159) *=* 2.48, *p* = 0.015; *t*(186) = 3.29, *p* = 0.002), increased right angular gyrus-right orbital frontal cortex (OFC) connection (*t*(159) *=* 2.93, *p* = 0.007; *t*(186) = 4.07, *p* = 0.001), increased left MTG-left anterior cingulate gyrus connection (*t*(159) *=* 4.02, *p* = 0.001; *t*(186) = 2.52, *p* = 0.013), 4 in total, see yellow line in Figure 1; altered connections shared by comorbidity and OCD were not found.

Altered connections unique to the comorbidity include increased connections including left OFC-left cerebellum_crus1 (*t*(159) *=* 3.28, *p* = 0.006), left Rolandic operculum-left cerebellum_crus1 (*t*(159) *=* 2.77, *p* = 0.009), right rectus-left cerebellum_crus1 (*t*(159) *=* 2.77, *p* = 0.009), right parahippocampal gyrus-left cerebellum_crus1 (*t*(159) *=* 2.51, *p* = 0.015), left fusiform gyrus (FG)-left cerebellum_crus1 (*t*(159) = 3.01, *p* = 0.007), right FG-left cerebellum_crus1 (*t*(159) *=* 2.45, *p* = 0.016), left Heschl gyrus-left cerebellum_crus1 (*t*(159) *=* 2.80, *p* = 0.009), left superior temporal gyrus (STG)-left cerebellum_crus1 (*t*(159) *=* 3.19, *p* = 0.006), right STG-left cerebellum_crus1 (*t*(159) *=* 3.16, *p* = 0.006), left cerebellum_crus2-left cerebellum_crusl (*t*(159) *=* 2.51, *p* = 0.015), 10 in total, see red line in Figure 1. These 10 connections were all significantly different for comorbidity vs. OCD and comorbidity vs. MDD (*p* < 0.05, FDR corrected, Table 3).

## 4. Discussion

In this study, we investigated the SCN alterations in patients with comorbid OCD and MDD. The results revealed that comorbidity of OCD and MDD showed both common and unique alterations compared with OCD or MDD alone. The unique covariant connection alterations were concentrated between the left cerebellum_crus1 and other brain regions, highlighting the important role of the cerebellum, especially cerebellum_crus1 region, in the neural basis of comorbid OCD and MDD.

The cerebellum is commonly acknowledged for its role in physical balance and motor control. However, in the last decade, its non-motor functions, such as emotional and cognitive functions, including emotion recognition and regulation, social cognition, decision making, and behavioral choice, have also been observed. This has led to a growing scientific interest in understanding how the cerebellum contributes to the pathology of psychiatric disorders [35, 36]. For example, research has found prefronto-cerebellar circuit structural abnormalities may be related to emotional and cognitive function deficits in MDD and connectivity analyses have shown reduced cerebro-cerebellar coupling of lobules VI and VIIA/B with prefrontal, posterior parietal, and limbic regions in patients with MDD [37, 38]. Some studies have also found OCD patients showed significantly increased fraction of amplitude of low-frequency fluctuation (fALFF) values in bilateral cerebellar and weakened connectivity among the left cerebellum Crus II, lobule VIII, and right striatum and which were associated with symptom severity [39]. Notably, recent neuroimaging studies have further investigated the distinct functional subregions in the cerebellum. Based on these results, the cerebellum_crus1 is mainly involved in the recognition, understanding during the social cognition processing, such as the understanding of others' intentions and the process of empathy [40].

In the comorbidity group of our study, there were a total of 10 unique altered covariant connections connected to the cerebellum_crus1, including left orbital OFC, left Rolandic operculum, right rectus, right parahippocampal gyrus, left FG, right FG, left Heschl gyrus, left STG, right STG, and left cerebelum_crus2. The OFC is a core area associated with emotion regulation. Its importance in emotional processing lies in its representation of the reward or affective value of taste, touch, texture, and facial expressions, and it learns to associate these stimuli with other factors to create representations of the expected reward value [41, 42]. The Rolandic operculum is crucial in integrating exteroceptive and interoceptive signals [43]. The rectus gyrus, located on the underside of the frontal lobe and medial to the OFC, is associated with temporal and spatial memory function [44]. The parahippocampal gyrus is situated below and medial to the occipital and temporal lobes. It plays a crucial role in transforming memory and processing spatial information, which is closely linked to cognition and emotion. The FG is generally considered a key structure for high-level vision, such as face perception and facial emotion processing [45, 46]. It has also been implicated in mental disorders, as shown in our previous study where FG was found to be associated with consummatory anhedonia in OCD [47]. The Heschl gyrus, which includes the primary auditory cortex [48]. The STG has two main functions: language processing and social perception. Furthermore, the STG plays a role in mentalizing, particularly when we are conscious of or hypothesize about the intentions or goal-directed behavior of ourselves or others [49–51], making it a region closely related to mental disorders. The cerebellum_crus2 plays a role in emotional expression and regulation, particularly in behavioral choices and decision-making processes during social interactions [40]. As a whole, the functions of the regions connected with the cerebellum_crus1 can be categorized into three main types: sensory transmission (e.g., FG and Heschl gyrus); information integration and evaluation (e.g., OFC, Rolandic operculum, STG, and cerebellum_crus2); and temporal and spatial memory processing (e.g., rectus gyrus and parahippocampal gyrus). These findings may indicate that the alterations in the cerebellum_crus1-related connections may be associated with wide range of cognitive and emotional processing in comorbidity. The findings emphasized the possible hub role of cerebellum in understanding the comorbidity and also underscored the critical role of broad cognitive and emotional processing in the occurrence and development of comorbidity. In addition, the SCN reflects morphological correlation over a large time scale, suggesting that comorbidity may have stable or trait alterations in the coordination of gray matter structure between cerebellum and other brain regions.

Regarding the mechanism of comorbidity of OCD and MDD, the prevailing view has been that MDD is a consequence of the burden and impairment caused by OCD [8, 52]. This perspective proposes that the neural basis of comorbidity may simply reflect a combination of features from both OCD and MDD. However, our study identified unique neuroimaging alterations in comorbidity that differed from OCD and MDD alone, suggesting that comorbidity may represent an independent disease type. This is in line with a previous genetic study which suggested the co-occurrence of OCD and MDD was primarily due to shared genetic factors rather than a causal relationship [53]. Looking forward, mathematical modeling methods (e.g., multilayer linear models, cross-lag model, and structural equation models) can be applied to integrate genetic methods (such as gene polymorphism detection) with multimodal neuroimaging techniques, thereby exploring the neurobiological basis of comorbidity.

Our study also found several altered connections that were shared by comorbidity and single diagnostic disorders. Altered connections shared by three groups was right MTG-left anterior cingulate gyrus; altered connections shared by comorbidity and MDD were right inferior parietal lobe-left anterior cingulate gyrus, right inferior parietal lobe-left posterior cingulated gyrus, right angular gyrus-right OFC, and left MTG-left anterior cingulate gyrus. These brain regions are also common brain regions associated with cognition or emotion. For instance, the MTG is a brain region associated with language, emotion, memory, and social cognition [54–56]. Studies have shown that the ALFF values of MTG were related to the BDI scores in MDD, while increased activation of MTG was related to guilt in OCD, and guilt is often a shared symptom of OCD and MDD [57, 58]. The cingulate gyrus plays a crucial role in the limbic system. Specifically, the anterior cingulate cortex (ACC) receives inputs from the OFC and amygdala, which in turn receive inputs from ventral stream areas. The posterior cingulate cortex (PCC) receives inputs from dorsal stream areas, including the parietal cortex, and has connections to the hippocampal memory system. The cingulate gyrus is involved in three important functions: emotion, action–outcome learning, and memory [59]. Some studies have found that ACC volume was significantly negatively associated with depressive Symptomatology Self-Report scores, and OCD patients showed higher activation in bilateral cingulate gyrus during symptom provocation [60, 61]. The angular gyrus, part of the inferior parietal lobule (IPL), is involved in semantic processing, word reading and comprehension, number processing, the default mode network, memory retrieval, attention and spatial cognition, reasoning, and social cognition [62, 63]. In MDD, studies have revealed angular gyrus-centered functional connectivity could predict Snaith-Hamilton Pleasure Scale scores, and in OCD, there are study found that the increased ALFF values of the right angular gyrus [64, 65]. Overall, these shared brain regions have been reported in OCD and MDD, which might be the neural basis for the overlapping symptom manifestations (such as obsessive thoughts and rumination) between OCD and MDD. These findings also confirm that comorbidity shares some of the same neural basis as OCD or MDD alone, indicating their potential importance in the occurrence and development of comorbidity.

Overall, our findings indicate that the neural basis of comorbidity extends beyond the mere superposition of single diagnostic diseases, providing a new theoretical direction for understanding and treating patients with comorbid OCD and MDD. In terms of treatment, we may need to differentiate comorbidity from single OCD or MDD. For instance, we should pay more attention to observing the therapeutic response of the drugs, and place greater emphasis on the combined use of drugs or the use of adequate doses and full treatment courses. And we may further focus on the cerebellum as the key area for targeted physical treatment. In terms of research direction, we may need to pay more attention to the uniqueness of comorbidity compared to single OCD or MDD, and consider the cerebellum as the core brain region in the mechanism of comorbidity of OCD and MDD for further in-depth exploration.

Several limitations in this study must be acknowledged. First, owing to the cross-sectional design, our research findings cannot explain causality and lacked follow-up data on the clinical progression of patients (such as potential diagnostic transitions), so we may not be able to fully elucidate the detailed mechanism of comorbidity. Therefore, in future research, we can track the changes in clinical symptoms and brain imaging features over multiple time points to explore the specific occurrence and development process of comorbidity. Second, the SCN in this study is constructed at the group level and cannot be directly correlated with clinical variables. Future studies may attempt to construct the SCN at the individual level to address this limitation. Third, the effect of medication could not be completely ruled out, although the medication status was controlled as covariates in the comparison between patient groups. In future studies, we aim to identify more effective statistical methods to eliminate the confounding effects of medication. Fourth, single-center study, participants' age ranged 18–35 years and inconsistent correction (only post-hoc *t*-tests were FDR-corrected while the initial F-tests remained uncorrected) may restrict the generalizability of the findings. Therefore, more optimal research design and consistent correction strategies will be taken into consideration in future studies to confirm our results.

In conclusion, our results suggested patients with comorbid OCD and MDD showed both common and unique altered covariant connections of the gray matter structure compared with OCD or MDD alone. The unique covariant connection alterations predominantly involved increased connections between the cerebellum and other brain regions, highlighting the important role of the cerebellum, especially the cerebellum_crus1 in the neural basis of comorbid OCD and MDD.

## Figures and Tables

**Table 1:** Demographic and clinical characteristics of OCD, MDD, comorbidity, and HC.

Variable	OCD (G1; *N* = 89)	MDD (G2; *N* = 94)	Comorbidity (G3; *N* = 67)	HC (G4; *N* = 94)	*F/H/U/χ* ^2^	*p*	Post hoc test (*p* < 0.05)
Age (years)	22.39 (4.88)	21.51 (4.43)	21.30 (4.10)	21.57 (3.42)	2.67	0.445	—
Sex (male,%)	45 (47.90)	30 (31.90)	30 (44.80)	45 (47.90)	11.30	0.010	—
Verbal intelligence	49.04 (8.40)	48.49 (7.96)	50.06 (7.99)	51.17 (6.16)	6.56	0.087	—
Age onset (years)	18.87 (4.57)	20.04 (4.73)	19.23 (4.23)	—	4.62	0.097	—
YBOCS	21.02 (5.88)	—	19.97 (7.57)	—	2810.50	0.540	—
YBOCS-O	10.94 (3.16)	—	11.42 (3.64)	—	2676.00	0.271	—
YBOCS-C	10.08 (3.89)	—	8.55 (4.96)	—	2485.50	0.075	—
STAI-T	56.57 (8.20)	61.91 (8.72)	63.28 (9.09)	40.81 (8.61)	177.41	<0.001	G1&G2&G3 >G4;G2&G3 >G1
STAI-S	52.29 (11.41)	59.14 (10.46)	60.42 (12.02)	37.49 (8.87)	150.02	<0.001	G1&G2&G3 >G4;G2&G3 >G1
BDI	17.26 (8.00)	30.29 (10.04)	32.57 (11.58)	6.35 (5.64)	212.34	<0.001	G1&G2&G3 >G4;G2&G3 >G1
Medication (%)	43 (48.3)	32 (34.0)	26 (38.8)	—	3.96	0.138	—
TIV	1565.31 (134.28)	1492.25 (121.86)	1539.73 (129.87)	1544.08 (128.77)	5.30	<0.001	G1&G4 >G2

*Note:* G1, OCD; G2, MDD; G3, comorbidity; G4, HC. The percentage of female subjects in MDD was higher than in OCD, *p* < 0.05 with Bonferroni correction.

Abbreviations: M, mean; SD, standard deviation; TIV, total intracranial volume; Y-BOCS, Yale-Brown Obsessive–Compulsive Scale; Y-BOCS-C, Yale-Brown Obsessive–Compulsive Scale-compulsion score; YBOCS-O, Yale-Brown Obsessive–Compulsive Scale-obsession score.

**Table 2:** Post-hoc *t*-test of SCN between patient groups and HC.

Comorbidity vs. HC				OCD vs. HC				MDD vs. HC			
AAL atlas	AAL atlas	*t*	*p* (FDR correction)	AAL atlas	AAL atlas	*t*	*p* (FDR correction)	AAL atlas	AAL atlas	*t*	*p* (FDR correction)
Frontal_Mid_Orb_L	Cerebelum_Crus1_L	3.28	0.006	Frontal_Mid_L	Frontal_Inf_Tri_R	−3.99	<0.001	Frontal_Sup_Orb_R	Precuneus_L	3.58	0.001
Rolandic_Oper_L	Cerebelum_Crus1_L	2.77	0.009	Frontal_Inf_Orb_R	Cerebelum_Crus1_L	−2.71	0.008	Parietal_Inf_R	Cingulum_Ant_L	3.74	0.001
Rectus_R	Cerebelum_Crus1_L	2.77	0.009	Cingulum_Post_L	Vermis_10	4.23	<0.001	Parietal_Inf_R	Cingulum_Post_L	3.29	0.002
ParaHippocampal_R	Cerebelum_Crus1_L	2.51	0.015	Heschl_L	Frontal_Mid_L	3.12	0.003	Parietal_Inf_R	Precuneus_L	3.70	0.001
Fusiform_L	Cerebelum_Crus1_L	3.01	0.007	Temporal_Sup_L	Frontal_Mid_L	2.51	0.013	Angular_R	Frontal_Med_Orb_R	4.07	0.001
Fusiform_R	Cerebelum_Crus1_L	2.45	0.016	Temporal_Mid_R	Cingulum_Ant_L	2.73	0.008	Heschl_L	Frontal_Mid_L	3.70	0.001
Parietal_Inf_R	Cingulum_Ant_L	2.96	0.007	Temporal_Inf_R	Temporal_Pole_Mid_R	3.94	<0.001	Temporal_Sup_L	Frontal_Mid_L	3.28	0.002
Parietal_Inf_R	Cingulum_Post_L	2.48	0.015	Cerebelum_8_L	Temporal_Pole_Mid_R	3.75	<0.001	Temporal_Mid_L	Cingulum_Ant_L	2.52	0.013
Angular_R	Frontal_Med_Orb_R	2.93	0.007	Cerebelum_8_R	Temporal_Pole_Mid_R	4.24	<0.001	Temporal_Mid_R	Cingulum_Ant_L	3.11	0.003
Heschl_L	Cerebelum_Crus1_L	2.80	0.009	Vermis_6	Temporal_Pole_Mid_R	3.53	0.001	Temporal_Inf_R	Temporal_Pole_Mid_R	3.16	0.003
Temporal_Sup_L	Cerebelum_Crus1_L	3.19	0.006	Vermis_7	Temporal_Pole_Mid_R	4.01	<0.001	Cerebelum_8_R	Temporal_Pole_Mid_R	2.77	0.007
Temporal_Sup_R	Cerebelum_Crus1_L	3.16	0.006	—	—	—	—	—	—	—	—
Temporal_Mid_L	Cingulum_Ant_L	4.02	0.001	—	—	—	—	—	—	—	—
Temporal_Mid_R	Cingulum_Ant_L	3.61	0.003	—	—	—	—	—	—	—	—
Cerebelum_Crus2_L	Cerebelum_Crus1_L	2.51	0.015	—	—	—	—	—	—	—	—

*Note:* There were 15 connections with significant differences between comorbidity and HC, 11 connections with significant differences between OCD and HC, 11 connections with significant differences between MDD and HC, all *p* < 0.05 with FDR correction.

**Table 3:** Comparison of altered connections unique to comorbidity in comorbidity vs. OCD and comorbidity vs. MDD.

AAL116 atlas	Comorbidity vs OCD		Comorbidity vs MDD	
	*t*	*p* (FDR correction)	*t*	*p* (FDRcorrection)
Cerebelum_Crus1_L-Frontal_Mid_Orb_L	4.65	<0.001	4.88	<0.001
Cerebelum_Crus1_L-Rolandic_Oper_L	3.93	<0.001	3.40	0.001
Cerebelum_Crus1_L-Rectus_R	4.27	<0.001	4.28	<0.001
Cerebelum_Crus1_L-ParaHippocampal_R	3.60	0.001	4.63	<0.001
Cerebelum_Crus1_L-Fusiform_L	3.26	0.002	5.16	<0.001
Cerebelum_Crus1_L-Fusiform_R	3.62	0.001	4.34	<0.001
Cerebelum_Crus1_L-Heschl_L	3.86	<0.001	3.05	0.003
Cerebelum_Crus1_L-Temporal_Sup_L	3.45	0.001	3.80	<0.001
Cerebelum_Crus1_L-Temporal_Sup_R	3.37	0.001	4.23	<0.001
Cerebelum_Crus1_L-Cerebelum_Crus2_L	2.09	0.039	4.06	<0.001

*Note:* There were 10 altered connections unique to comorbidity, all *p* < 0.05 with FDR correction.

## Data Availability

The data supporting the results of the study are available upon request from the corresponding author. The data are not publicly available due to privacy or ethical restrictions.

## Supporting Information

Supporting Information Table S1: Normality tests of demographic and clinical characteristics of OCD, MDD, comorbidity, and HC. Table S2: Test of homogeneity of variance of demographic and clinical characteristics of OCD, MDD, comorbidity, and HC. Table S3: ACNOVA analysis of SCN among four groups. Table S4: Post-hoc *t*-test of SCN between patient groups and HC (uncorrected).

## References

[1] Millet B., Kochman F., Gallarda T. (2004). Phenomenological and Comorbid Features Associated in Obsessive-Compulsive Disorder: Influence of Age of Onset. *Journal of Affective Disorders*.

[2] Pinto A., Mancebo M. C., Eisen J. L., Pagano M. E., Rasmussen S. A. (2006). The Brown Longitudinal Obsessive Compulsive Study: Clinical Features and Symptoms of the Sample at Intake. *The Journal of Clinical Psychiatry*.

[3] Lochner C., Fineberg N. A., Zohar J. (2014). Comorbidity in Obsessive-Compulsive Disorder (OCD): A Report From the International College of Obsessive-Compulsive Spectrum Disorders (ICOCS). *Comprehensive Psychiatry*.

[4] Rickelt J., Viechtbauer W., Lieverse R. (2016). The Relation Between Depressive and Obsessive-Compulsive Symptoms in Obsessive-Compulsive Disorder: Results From a Large, Naturalistic Follow-Up Study. *Journal of Affective Disorders*.

[5] Torres A. R., Ramos-Cerqueira A. T., Ferrão Y. A., Fontenelle L. F., do Rosário M. C., Miguel E. C. (2011). Suicidality in Obsessive-Compulsive Disorder: Prevalence and Relation to Symptom Dimensions and Comorbid Conditions. *The Journal of Clinical Psychiatry*.

[6] Kamath P., Reddy Y. C., Kandavel T. (2007). Suicidal Behavior in Obsessive-Compulsive Disorder. *The Journal of Clinical Psychiatry*.

[7] Brown H. M., Lester K. J., Jassi A., Heyman I., Krebs G. (2015). Paediatric Obsessive-Compulsive Disorder and Depressive Symptoms: Clinical Correlates and CBT Treatment Outcomes. *Journal of Abnormal Child Psychology*.

[8] Storch E. A., Abramowitz J. S., Keeley M. (2009). Correlates and Mediators of Functional Disability in Obsessive-Compulsive Disorder. *Depression and Anxiety*.

[9] Alexander G. E., DeLong M. R., Strick P. L. (1986). Parallel Organization of Functionally Segregated Circuits Linking Basal Ganglia and Cortex. *Annual Review of Neuroscience*.

[10] Milad M. R., Rauch S. L. (2012). Obsessive-Compulsive Disorder: Beyond Segregated Cortico-Striatal Pathways. *Trends in Cognitive Sciences*.

[11] Stein D. J., Costa D. L. C., Lochner C. (2019). Obsessive-Compulsive Disorder. *Nature Reviews Disease Primers*.

[12] Phelps E. A. (2004). Human Emotion and Memory: Interactions of the Amygdala and Hippocampal Complex. *Current Opinion in Neurobiology*.

[13] Tovote P., Fadok J. P., Lüthi A. (2015). Neuronal Circuits for Fear and Anxiety. *Nature Reviews Neuroscience*.

[14] Haber S. N., Knutson B. (2010). The Reward Circuit: Linking Primate Anatomy and Human Imaging. *Neuropsychopharmacology*.

[15] Nestler E. J., Barrot M., DiLeone R. J., Eisch A. J., Gold S. J., Monteggia L. M. (2002). Neurobiology of Depression. *Neuron*.

[16] Cardoner N., Soriano-Mas C., Pujol J. (2007). Brain Structural Correlates of Depressive Comorbidity in Obsessive-Compulsive Disorder. *NeuroImage*.

[17] Saxena S., Brody A. L., Ho M. L. (2001). Cerebral Metabolism in Major Depression and Obsessive-Compulsive Disorder Occurring Separately and Concurrently. *Biological Psychiatry*.

[18] Anholt G. E., Aderka I. M., van Balkom A. J. (2011). The Impact of Depression on the Treatment of Obsessive-Compulsive Disorder: Results From a 5-Year Follow-Up. *Journal of Affective Disorders*.

[19] Huppert J. D., Simpson H. B., Nissenson K. J., Liebowitz M. R., Foa E. B. (2009). Quality of Life and Functional Impairment in Obsessive-Compulsive Disorder: A Comparison of Patients With and Without Comorbidity, Patients in Remission, and Healthy Controls. *Depression and Anxiety*.

[20] Chasson G. S., Bello M. S., Luxon A. M., Graham T. A. A., Leventhal A. M. (2017). Transdiagnostic Emotional Vulnerabilities Linking Obsessive-Compulsive and Depressive Symptoms in a Community-Based Sample of Adolescents. *Depression and Anxiety*.

[21] Shamsher Khan R. M., Muneer A., Nawaz K. (2018). Frequency of Obsessive Compulsive Symptoms in Depression: A Hospital-Based Cross-Sectional Study. *JPMA The Journal of the Pakistan Medical Association*.

[22] Riesel A., Klawohn J., Grützmann R. (2019). Error-Related Brain Activity as a Transdiagnostic Endophenotype for Obsessive-Compulsive Disorder, Anxiety and Substance use Disorder. *Psychological Medicine*.

[23] Alexander-Bloch A., Giedd J. N., Bullmore E. (2013). Imaging Structural Co-Variance Between Human Brain Regions. *Nature Reviews Neuroscience*.

[24] Yun J. Y., Jang J. H., Kim S. N., Jung W. H., Kwon J. S. (2015). Neural Correlates of Response to Pharmacotherapy in Obsessive-Compulsive Disorder: Individualized Cortical Morphology-Based Structural Covariance. *Progress in Neuro-Psychopharmacology & Biological Psychiatry*.

[25] Evans A. C. (2013). Networks of Anatomical Covariance. *NeuroImage*.

[26] Draganski B., Gaser C., Busch V., Schuierer G., Bogdahn U., May A. (2004). Neuroplasticity: Changes in Grey Matter Induced by Training. *Nature*.

[27] Pezawas L., Verchinski B. A., Mattay V. S. (2004). The Brain-Derived Neurotrophic Factor val66Met Polymorphism and Variation in Human Cortical Morphology. *The Journal of Neuroscience*.

[28] Subirà M., Cano M., de Wit S. J. (2016). Structural Covariance of Neostriatal and Limbic Regions in Patients With Obsessive-Compulsive Disorder. *Journal of Psychiatry & Neuroscience*.

[29] Zhang X., Xie M., Li W. (2024). Abnormalities of Structural Covariance of Insular Subregions in Drug-Naïve OCD Patients. *Cerebral Cortex (New York, NY: 1991)*.

[30] Watanabe K., Kakeda S., Katsuki A. (2020). Whole-Brain Structural Covariance Network Abnormality in First-Episode and Drug-Naïve Major Depressive Disorder. *Psychiatry Research Neuroimaging*.

[31] Han S., Zheng R., Li S. (2023). Altered Structural Covariance Network of Nucleus Accumbens Is Modulated by Illness Duration and Severity of Symptom in Depression. *Journal of Affective Disorders*.

[32] Beck A. T., Ward C. H., Mendelson M., Mock J., Erbaugh J. (1961). An Inventory for Measuring Depression. *Archives of General Psychiatry*.

[33] Shahid A., Wilkinson K., Marcu S., Shapiro C. M. (2011). *State-Trait Anxiety Inventory (STAI)*.

[34] Goodman W. K., Price L. H., Rasmussen S. A. (1989). The Yale-Brown Obsessive Compulsive Scale. I. Development, use, and Reliability. *Archives of General Psychiatry*.

[35] Diedrichsen J., King M., Hernandez-Castillo C., Sereno M., Ivry R. B. (2019). Universal Transform or Multiple Functionality? Understanding the Contribution of the Human Cerebellum Across Task Domains. *Neuron*.

[36] Schmahmann J. D., Guell X., Stoodley C. J., Halko M. A. (2019). The Theory and Neuroscience of Cerebellar Cognition. *Annual Review of Neuroscience*.

[37] Depping M. S., Schmitgen M. M., Kubera K. M., Wolf R. C. (2018). Cerebellar Contributions to Major Depression. *Frontiers in Psychiatry*.

[38] Minichino A., Bersani F. S., Trabucchi G. (2014). The Role of Cerebellum in Unipolar and Bipolar Depression: A Review of the Main Neurobiological Findings. *Rivista di Psichiatria*.

[39] Zhang H., Wang B., Li K. (2019). Altered Functional Connectivity Between the Cerebellum and the Cortico-Striato-Thalamo-Cortical Circuit in Obsessive-Compulsive Disorder. *Frontiers in Psychiatry*.

[40] Gao Z., Liu X., Zhang D., Liu M., Hao N. (2020). The Indispensable Role of the Cerebellum in Visual Divergent Thinking. *Scientific Reports*.

[41] Rolls E. T. (2014). Emotion and Decision-Making Explained: A Précis. *Cortex; A Journal Devoted to the Study of the Nervous System and Behavior*.

[42] Rolls E. T. (2019). The Orbitofrontal Cortex and Emotion in Health and Disease, Including Depression. *Neuropsychologia*.

[43] Blefari M. L., Martuzzi R., Salomon R. (2017). Bilateral Rolandic Operculum Processing Underlying Heartbeat Awareness Reflects Changes in Bodily Self-Consciousness. *The European Journal of Neuroscience*.

[44] Ballmaier M., Toga A. W., Blanton R. E. (2004). Anterior Cingulate, Gyrus Rectus, and Orbitofrontal Abnormalities in Elderly Depressed Patients: An MRI-Based Parcellation of the Prefrontal Cortex. *American Journal of Psychiatry*.

[45] Fonville L., Giampietro V., Surguladze S., Williams S., Tchanturia K. (2014). Increased BOLD Signal in the Fusiform Gyrus During Implicit Emotion Processing in Anorexia Nervosa. *NeuroImage: Clinical*.

[46] Fusar-Poli P., Bhattacharyya S., Allen P. (2010). Effect of Image Analysis Software on Neurofunctional Activation During Processing of Emotional Human Faces. *Journal of Clinical Neuroscience*.

[47] Du H., Xia J., Fan J. (2022). Spontaneous Neural Activity in the Right Fusiform Gyrus and Putamen Is Associated With Consummatory Anhedonia in Obsessive Compulsive Disorder. *Brain Imaging and Behavior*.

[48] Takahashi T., Yücel M., Lorenzetti V. (2010). An MRI Study of the Superior Temporal Subregions in Patients With Current and past Major Depression. *Progress in Neuro-Psychopharmacology & Biological Psychiatry*.

[49] Allison T., Puce A., McCarthy G. (2000). Social Perception From Visual Cues: Role of the STS Region. *Trends in Cognitive Sciences*.

[50] Hoekert M., Bais L., Kahn R. S., Aleman A. (2008). Time Course of the Involvement of the Right Anterior Superior Temporal Gyrus and the Right Fronto-Parietal Operculum in Emotional Prosody Perception. *PLoS One*.

[51] Sander D., Grandjean D., Pourtois G. (2005). Emotion and Attention Interactions in Social Cognition: Brain Regions Involved in Processing Anger Prosody. *NeuroImage*.

[52] Storch E. A., Lewin A. B., Larson M. J., Geffken G. R., Murphy T. K., Geller D. A. (2012). Depression in Youth With Obsessive-Compulsive Disorder: Clinical Phenomenology and Correlates. *Psychiatry Research*.

[53] Bolhuis K., McAdams T. A., Monzani B. (2014). Aetiological Overlap Between Obsessive-Compulsive and Depressive Symptoms: A Longitudinal Twin Study in Adolescents and Adults. *Psychological Medicine*.

[54] Giraud A. L., Kell C., Thierfelder C. (2004). Contributions of Sensory Input, Auditory Search and Verbal Comprehension to Cortical Activity During Speech Processing. *Cerebral cortex (New York, NY: 1991)*.

[55] Hesling I., Clément S., Bordessoules M., Allard M. (2005). Cerebral Mechanisms of Prosodic Integration: Evidence From Connected Speech. *NeuroImage*.

[56] Whitney C., Jefferies E., Kircher T. (2011). Heterogeneity of the Left Temporal Lobe in Semantic Representation and Control: Priming Multiple Versus Single Meanings of Ambiguous Words. *Cerebral Cortex (New York, NY: 1991)*.

[57] Cheng C., Dong D., Jiang Y. (2019). State-Related Alterations of Spontaneous Neural Activity in Current and Remitted Depression Revealed by Resting-State fMRI. *Frontiers in Psychology*.

[58] Gonçalves Ó.F, Carvalho S., Leite J., Fernandes-Gonçalves A., Carracedo A., Sampaio A. (2016). Cognitive and Emotional Impairments in Obsessive-Compulsive Disorder: Evidence From Functional Brain Alterations. *Porto Biomedical Journal*.

[59] Rolls E. T. (2019). The Cingulate Cortex and Limbic Systems for Emotion, Action, and Memory. *Brain Structure & Function*.

[60] Ibrahim H. M., Kulikova A., Ly H., Rush A. J., Sherwood Brown E. (2022). Anterior Cingulate Cortex in Individuals With Depressive Symptoms: A Structural MRI Study. *Psychiatry Research Neuroimaging*.

[61] Yu J., Zhou P., Yuan S. (2022). Symptom Provocation in Obsessive-Compulsive Disorder: A Voxel-Based Meta-Analysis and Meta-Analytic Connectivity Modeling. *Journal of Psychiatric Research*.

[62] Seghier M. L. (2013). The Angular Gyrus: Multiple Functions and Multiple Subdivisions. *The Neuroscientist: A Review Journal Bringing Neurobiology, Neurology and Psychiatry*.

[63] Gray O., Fry L., Montaldi D. (2020). Information Content Best Characterises the Hemispheric Selectivity of the Inferior Parietal Lobe: A Meta-Analysis. *Scientific Reports*.

[64] Kang L., Wang W., Zhang N. (2023). Anhedonia and Dysregulation of an Angular Gyrus-Centred and Dynamic Functional Network in Adolescent-Onset Depression. *Journal of Affective Disorders*.

[65] Yu X. M., Qiu L. L., Huang H. X. (2021). Comparison of Resting-State Spontaneous Brain Activity Between Treatment-Naive Schizophrenia and Obsessive-Compulsive Disorder. *BMC Psychiatry*.

