# Supplementary material for: Shared and Unique Structural Covariance Connectivity in Comorbidity of Obsessive-Compulsive Disorder and Major Depressive Disorder
Source: Depress Anxiety. 2025 Nov 26;2025:1087782. doi: 10.1155/da/1087782 (PMC12674874; doi:10.1155/da/1087782)
Supplement: Supporting Information — Table S1: Normality tests of demographic and clinical characteristics of OCD, MDD, comorbidity, and HC. Table S2: Test of homogeneity of variance of demographic and clinical characteristics of OCD, MDD, comorbidity, and HC. Table S3: ACNOVA analysis of SCN among four groups. Table S4: Post-hoc t-test of SCN between patient groups and HC (uncorrected). [file 1087782.f1.docx]

Table S1 Normality tests of demographic and clinical characteristics of OCD, MDD, comorbidity and HC

|  | OCD  (G1; N=89) | MDD  (G2;N=94) | Comorbidity  (G3;N=67) | HC  (G4;N=94) |
| --- | --- | --- | --- | --- |
| Age(years) | *D=0.14, P<0.001* | *D=0.20, P<0.001* | *D=0.17, P<0.001* | *D=0.25, P<0.001* |
| sex(male,%) |  |  |  |  |
| verbal intelligence | *D=0.10, P=0.039* | *D=0.07, P=0.200* | *D=0.09, P=0.200* | *D=0.11, P=0.010* |
| Age onset(years) | *D=0.15, P<0.001* | *D=0.46, P<0.001* | *D=0.19, P=0.000* |  |
| YBOCS | *D=0.09, P=0.051* |  | *D=0.09, P=0.200* |  |
| YBOCS-O | *D=0.17, P<0.001* |  | *D=0.14, P=0.003* |  |
| YBOCS-C | *D=0.09, P=0.049* |  | *D=0.10, P=0.091* |  |
| STAI-T | *D=0.07, P=0.200* | *D=0.07, P=0.200* | *D=0.11, P=0.033* | *D=0.07, P=0.200* |
| STAI-S | *D=0.10, P=0.030* | *D=0.06, P=0.200* | *D=0.09, P=0.200* | *D=0.07, P=0.200* |
| BDI | *D=0.07, P=0.200* | *D=0.08, P=0.176* | *D=0.06, P=0.200* | *D=0.15, P<0.001* |
| Medication(%) |  |  |  |  |
| TIV | *D=0.06, P=0.200* | *D=0.06, P=0.200* | *D=0.09, P=0.200* | *D=0.05, P=0.200* |

Note: G1，OCD；G2，MDD；G3，comorbidity；G4, HC；Y-BOCS，Yale-Brown Obsessive–Compulsive Scale；YBOCS-O，Yale-Brown Obsessive–Compulsive Scale-obsession score；Y-BOCS-C，Yale-Brown Obsessive–Compulsive Scale-compulsion score；TIV，total intracranial volume.

Table S2 Test of Homogeneity of Variance of demographic and clinical characteristics of OCD, MDD, comorbidity and HC（*M(SD)*）

|  | OCD  (G1; N=89) | MDD  (G2;N=94) | Comorbidity  (G3;N=67) | HC  (G4;N=94) | *F* | *p* |
| --- | --- | --- | --- | --- | --- | --- |
| Age(years) | 22.39(4.88) | 21.51(4.43) | 21.30(4.10) | 21.57(3.42) | 4.29 | 0.005 |
| sex(male,%) | 45(47.90) | 30(31.90) | 30(44.80) | 45(47.90) |  |  |
| verbal intelligence | 49.04(8.40) | 48.49(7.96) | 50.06(7.99) | 51.17(6.16) | 2.99 | 0.031 |
| Age onset(years) | 18.87(4.57) | 20.04(4.73) | 19.23(4.23) |  | 2.90 | 0.057 |
| YBOCS | 21.02(5.88) |  | 19.97(7.57) |  | 6.12 | 0.014 |
| YBOCS-O | 10.94(3.16) |  | 11.42(3.64) |  | 3.11 | 0.080 |
| YBOCS-C | 10.08(3.89) |  | 8.55(4.96) |  | 7.56 | 0.007 |
| STAI-T | 56.57(8.20) | 61.91(8.72) | 63.28(9.09) | 40.81(8.61) | 0.22 | 0.881 |
| STAI-S | 52.29(11.41) | 59.14(10.46) | 60.42(12.02) | 37.49(8.87) | 2.73 | 0.044 |
| BDI | 17.26(8.00) | 30.29(10.04) | 32.57(11.58) | 6.35(5.64) | 12.69 | <0.001 |
| Medication(%) | 43(48.3) | 32(34.0) | 26(38.8) |  |  |  |
| TIV | 1565.31（134.28） | 1492.25（121.86） | 1539.73（129.87） | 1544.08（128.77） | 0.44 | 0.727 |

Note: M, mean；SD，standard deviation；G1，OCD；G2，MDD；G3，comorbidity；G4, HC；Y-BOCS，Yale-Brown Obsessive–Compulsive Scale；YBOCS-O，Yale-Brown Obsessive–Compulsive Scale-obsession score；Y-BOCS-C，Yale-Brown Obsessive–Compulsive Scale-compulsion score；TIV，total intracranial volume.

Table S3 ACNOVA analysis of SCN among four groups

| AAL atlas | | |  | AAL atlas | | *F* | | *p*(uncorrected) |  |
| --- | --- | --- | --- | --- | --- | --- | --- | --- | --- |
| Frontal_Sup_R | | - | | Cerebelum_Crus1_L | 5.71 | | <0.001 | | |
| Frontal_Sup_Orb_L | | - | | Cerebelum_Crus1_L | 5.84 | | <0.001 | | |
| Frontal_Sup_Orb_R | | - | | Precuneus_L | 5.69 | | <0.001 | | |
| Frontal_Mid_L | | - | | Frontal_Inf_Tri_R | 5.80 | | <0.001 | | |
| Frontal_Mid_Orb_L | | - | | Cerebelum_Crus1_L | 9.03 | | <0.001 | | |
| Frontal_Inf_Orb_L | | - | | Cerebelum_Crus1_L | 6.85 | | <0.001 | | |
| Frontal_Inf_Orb_R | | - | | Cerebelum_Crus1_L | 5.89 | | <0.001 | | |
| Rolandic_Oper_L | | - | | Cerebelum_Crus1_L | 6.10 | | <0.001 | | |
| Supp_Motor_Area_R | | - | | Cerebelum_Crus1_L | 7.11 | | <0.001 | | |
| Frontal_Sup_Medial_L | | - | | Cerebelum_Crus1_L | 5.64 | | <0.001 | | |
| Rectus_L | | - | | Cerebelum_Crus1_L | 6.04 | | <0.001 | | |
| Rectus_R | | - | | Cerebelum_Crus1_L | 7.25 | | <0.001 | | |
| Insula_L | | - | | Cerebelum_Crus1_L | 7.19 | | <0.001 | | |
| Insula_R | | - | | Cerebelum_Crus1_L | 6.03 | | <0.001 | | |
| Cingulum_Mid_L | | - | | Cerebelum_Crus1_L | 6.03 | | <0.001 | | |
| Cingulum_Post_L | | - | | Vermis_10 | 7.27 | | <0.001 | | |
| ParaHippocampal_R | | - | | Cerebelum_Crus1_L | 7.41 | | <0.001 | | |
| Fusiform_L | | - | | Cerebelum_Crus1_L | 7.86 | | <0.001 | | |
| Fusiform_R | | - | | Cerebelum_Crus1_L | 6.26 | | <0.001 | | |
| Parietal_Inf_R | | - | | Cingulum_Ant_L | 7.07 | | <0.001 | | |
| Parietal_Inf_R | | - | | Cingulum_Post_L | 6.49 | | <0.001 | | |
| Parietal_Inf_R | - | | Precuneus_L | 5.84 | | <0.001 | | |  |
| Angular_R | - | | Frontal_Med_Orb_R | 6.95 | | <0.001 | | |  |
| Heschl_L | - | | Frontal_Mid_L | 6.87 | | <0.001 | | |  |
| Heschl_L | - | | Cerebelum_Crus1_L | 6.15 | | <0.001 | | |  |
| Temporal_Sup_L | - | | Frontal_Mid_L | 7.01 | | <0.001 | | |  |
| Temporal_Sup_L | - | | Cerebelum_Crus1_L | 6.10 | | <0.001 | | |  |
| Temporal_Sup_R | - | | Cerebelum_Crus1_L | 6.01 | | <0.001 | | |  |
| Temporal_Mid_L | - | | Cingulum_Ant_L | 6.18 | | <0.001 | | |  |
| Temporal_Mid_R | - | | Cingulum_Ant_L | 5.69 | | <0.001 | | |  |
| Temporal_Inf_R | - | | Temporal_Pole_Mid_R | 7.14 | | <0.001 | | |  |
| Cerebelum_Crus2_L | - | | Cerebelum_Crus1_L | 6.29 | | <0.001 | | |  |
| Cerebelum_8_L | - | | Temporal_Pole_Mid_R | 6.26 | | <0.001 | | |  |
| Cerebelum_8_R | - | | Temporal_Pole_Mid_R | 6.60 | | <0.001 | | |  |
| Vermis_6 | - | | Temporal_Pole_Mid_R | 5.87 | | <0.001 | | |  |
| Vermis_7 | - | | Temporal_Pole_Mid_R | 5.91 | | <0.001 | | |  |

notes：there were 36 connections with significant differences, all *p*<0.001, uncorrected.

Table S4 Post-hoc *t*-test of SCN between patient groups and HC

| **Comorbidity vs HC** | | | | | **OCD vs HC** | | | | | **MDD vs HC** | | | | |  |
| --- | --- | --- | --- | --- | --- | --- | --- | --- | --- | --- | --- | --- | --- | --- | --- |
| AAL atlas | | AAL atlas | *t* | *p*(uncorrected) | AAL atlas |  | AAL atlas | *t* | *p*(uncorrected) | AAL atlas |  | AAL atlas | *t* | *p*(uncorrected) | |
| Frontal_Mid_Orb_L | - | Cerebelum_Crus1_L | 3.28 | 0.001 | Frontal_Mid_L | - | Frontal_Inf_Tri_R | -3.99 | <0.001 | Frontal_Sup_Orb_R | - | Precuneus_L | 3.58 | <0.001 | |
| Rolandic_Oper_L | - | Cerebelum_Crus1_L | 2.77 | 0.006 | Frontal_Inf_Orb_R | - | Cerebelum_Crus1_L | -2.71 | 0.007 | Parietal_Inf_R | - | Cingulum_Ant_L | 3.74 | <0.001 | |
| Rectus_R | - | Cerebelum_Crus1_L | 2.77 | 0.006 | Cingulum_Post_L | - | Vermis_10 | 4.23 | <0.001 | Parietal_Inf_R | - | Cingulum_Post_L | 3.29 | 0.001 | |
| ParaHippocampal_R | - | Cerebelum_Crus1_L | 2.51 | 0.013 | Heschl_L | - | Frontal_Mid_L | 3.12 | 0.002 | Parietal_Inf_R | - | Precuneus_L | 3.70 | <0.001 | |
| Fusiform_L | - | Cerebelum_Crus1_L | 3.01 | 0.003 | Temporal_Sup_L | - | Frontal_Mid_L | 2.51 | 0.013 | Angular_R | - | Frontal_Med_Orb_R | 4.07 | <0.001 | |
| Fusiform_R | - | Cerebelum_Crus1_L | 2.45 | 0.015 | Temporal_Mid_R | - | Cingulum_Ant_L | 2.73 | 0.006 | Heschl_L | - | Frontal_Mid_L | 3.70 | <0.001 | |
| Parietal_Inf_R | - | Cingulum_Ant_L | 2.96 | 0.003 | Temporal_Inf_R | - | Temporal_Pole_Mid_R | 3.94 | <0.001 | Temporal_Sup_L | - | Frontal_Mid_L | 3.28 | 0.001 | |
| Parietal_Inf_R | - | Cingulum_Post_L | 2.48 | 0.015 | Cerebelum_8_L | - | Temporal_Pole_Mid_R | 3.75 | <0.001 | Temporal_Mid_L | - | Cingulum_Ant_L | 2.52 | 0.012 | |
| Angular_R | - | Frontal_Med_Orb_R | 2.93 | 0.004 | Cerebelum_8_R | - | Temporal_Pole_Mid_R | 4.24 | <0.001 | Temporal_Mid_R | - | Cingulum_Ant_L | 3.11 | 0.002 | |
| Heschl_L | - | Cerebelum_Crus1_L | 2.80 | 0.006 | Vermis_6 | - | Temporal_Pole_Mid_R | 3.53 | <0.001 | Temporal_Inf_R | - | Temporal_Pole_Mid_R | 3.16 | 0.002 | |
| Temporal_Sup_L | - | Cerebelum_Crus1_L | 3.19 | 0.002 | Vermis_7 | - | Temporal_Pole_Mid_R | 4.01 | <0.001 | Cerebelum_8_R | - | Temporal_Pole_Mid_R | 2.77 | 0.006 | |
| Temporal_Sup_R | - | Cerebelum_Crus1_L | 3.16 | 0.002 |  |  |  |  |  |  |  |  |  |  | |
| Temporal_Mid_L | - | Cingulum_Ant_L | 4.02 | <0.001 |  |  |  |  |  |  |  |  |  |  | |
| Temporal_Mid_R | - | Cingulum_Ant_L | 3.61 | <0.001 |  |  |  |  |  |  |  |  |  |  | |
| Cerebelum_Crus2_L | - | Cerebelum_Crus1_L | 2.51 | 0.013 |  |  |  |  |  |  |  |  |  |  | |

notes：there were 15 connections with significant differences between comorbidity and HC, 11 connections with significant differences between OCD and HC, 11 connections with significant differences between MDD and HC.
